# Case Report: refractory hemophagocytic lymphohistiocytosis progression despite complete metabolic response in a patient with stage IV extranodal NK/T-cell lymphoma: clinical observation of “tumor-immune dissociation” phenomenon

**DOI:** 10.3389/fimmu.2026.1832423

**Published:** 2026-07-01

**Authors:** Haoyuan Huang, Chan Long, Fangying Zheng, Xiaoling Zhang

**Affiliations:** Department of Hematology, Huizhou First Hospital, Huizhou, China

**Keywords:** Epstein-Barr virus DNA, extranodal NK/T-cell lymphoma, hemophagocytic, immune biomarkers, lymphohistiocytosis, tumor-immune dissociation

## Abstract

Extranodal NK/T-cell lymphoma (ENKTCL) is an Epstein-Barr virus (EBV)-associated mature T- and NK-cell neoplasm with a notably high prevalence in Asian populations. It is a leading malignant cause of secondary hemophagocytic lymphohistiocytosis (HLH), a life-threatening immune hyperactivation syndrome associated with poor prognosis in ENKTCL patients. Current clinical guidelines lack evidence-based post-remission monitoring protocols for the early detection of HLH progression in ENKTCL patients who achieve complete metabolic response (CMR). We report the case of a 59-year-old woman with stage IVB extra-nasal ENKTCL and biopsy-confirmed secondary HLH at initial diagnosis, who achieved radiologic CMR following frontline HLH induction therapy and three cycles of lymphoma-specific chemotherapy. Despite persistent CMR, she developed refractory HLH progression, and ultimately died from progressive HLH and multiple organ failure. Serial dynamic monitoring of tumor biomarkers (plasma EBV-DNA), direct immune activation biomarkers (soluble CD25 [sCD25]), and HLH-specific disease activity biomarkers (serum ferritin, triglycerides) revealed a “tumor-immune dissociation” phenomenon—aberrant immune activation and HLH progression occurring independently of radiologic tumor progression, with sCD25 remaining persistently elevated throughout the disease course, though subclinical EBV reactivation or microscopic disease undetectable by PET/CT cannot be excluded. This case demonstrates that HLH can progress independently of radiologically detectable tumor burden even after achieving CMR, underscoring a critical limitation of current response-adapted follow-up strategies. The observed “tumor-immune dissociation” highlights the urgent need to incorporate immune activation biomarkers into routine surveillance for high-risk ENKTCL patients, as well as to conduct early evaluations of allogeneic hematopoietic stem cell transplantation for eligible individuals.

## 1 Introduction

Extranodal NK/T-cell lymphoma (ENKTCL) is an EBV-associated mature T- and NK-cell neoplasm with a notably high prevalence in East Asian populations. It is also one of the most common causes of malignancy-associated HLH (M-HLH) in Asia (1, 2). HLH is a life-threatening cytokine storm syndrome characterized by uncontrolled activation of cytotoxic T cells and macrophages (3). Previous multicenter studies have confirmed that ENKTCL-related HLH is associated with a one-year overall survival rate of less than 30%, posing a major clinical challenge in lymphoma management (4).

Current clinical guidelines primarily focus on the diagnosis and induction treatment of *de novo* ENKTCL and HLH. For post-remission follow-up of ENKTCL, routine surveillance strategies rely almost exclusively on whole-body PET/CT and plasma EBV-DNA quantification to monitor tumor recurrence. However, no standardized evidence-based recommendations exist for monitoring immune activation or HLH disease activity biomarkers directly related to HLH pathogenesis. Although HLH is a well-recognized and frequent complication of ENKTCL at initial presentation (5), its occurrence and progression in patients who have achieved complete metabolic response (CMR) remain virtually undescribed in the existing literature. To our knowledge, this is the first detailed case of refractory HLH progression despite sustained PET/CT-confirmed CMR in ENKTCL. We propose “tumor-immune dissociation” to describe this novel phenomenon. While no identical cases exist, existing studies confirm that malignancy-associated HLH can become self-sustaining via cytokine storm independent of tumor burden, especially in EBV-associated lymphomas (5). We also provide a comprehensive longitudinal profile of tumor and immune biomarkers not previously reported, with direct implications for optimizing post-remission surveillance and improving clinical outcomes in this high-risk patient population.

## 2 Case description

A 59-year-old Han Chinese woman from Guangdong Province presented to our hospital with a one-month history of a back mass and a two-week history of recurrent high fevers. One month prior to admission, she developed a tender back mass accompanied by nasal obstruction, rhinorrhea, and sore throat without any obvious precipitating factors and received no further medical evaluation at that time. Two weeks before admission, she experienced intermittent high fevers with a peak temperature of 39 °C, without accompanying headache, chest tightness, dyspnea, joint pain, skin rash, or other focal symptoms. She was initially treated empirically with ceftriaxone (2 g intravenously once daily for 5 days), symptomatic antipyretic therapy, and topical mupirocin ointment for the back lesion. However, her clinical symptoms did not improve, and repeated complete blood counts revealed persistent leukopenia. Her past medical history was notable for an 8-year history of primary hypertension, managed with regular oral amlodipine besylate (5 mg once daily) and well-controlled blood pressure. She had no history of malignancy, autoimmune disease, immunodeficiency, smoking, or alcohol consumption, and no relevant family history of hematologic malignancies or HLH-related genetic disorders.

At baseline admission, the patient was hemodynamically stable, with a body temperature of 36.5°C, a heart rate of 92 beats per minute, a respiratory rate of 17 breaths per minute, and blood pressure of 113/60 mmHg. Physical examination revealed a moderately anemic appearance and a 1.5 × 1.5 cm partially ulcerated and crusted mass on the back, without purulent discharge or palpable fluctuation. There was no superficial lymphadenopathy, no jaundice of the skin or sclera, no petechiae or ecchymosis, no sternal tenderness, and no hepatosplenomegaly was palpated beneath the costal margin. Initial laboratory workup showed bicytopenia, with hemoglobin 90 g/L, platelet 51 × 10^9^/L, and white blood cell 1.55 × 10^9^/L. Markedly elevated inflammatory and immune activation markers included serum ferritin 8,287 ng/mL, triglycerides 3.03 mmol/L, fibrinogen 1.3 g/L, lactate dehydrogenase 545 U/L, and C-reactive protein 47.29 mg/L. HLH-specific testing showed NK cell activity of 15.59% and soluble CD25 (sCD25) levels of 24,227 U/mL Notably, sCD25 and NK cell activity assays required send-out testing to a third-party reference laboratory, were not covered by medical insurance, and resulted in high out-of-pocket costs for the patient. Etiological testing showed a plasma EBV-DNA load of 2.32 × 10^5^ copies/mL, while tests for cytomegalovirus, human immunodeficiency virus, and other common viral pathogens were negative. Multiple sets of blood cultures showed no bacterial growth. The baseline HScore for adult HLH was calculated at 233 points, corresponding to a >90% probability of HLH.

**Figure 1:**
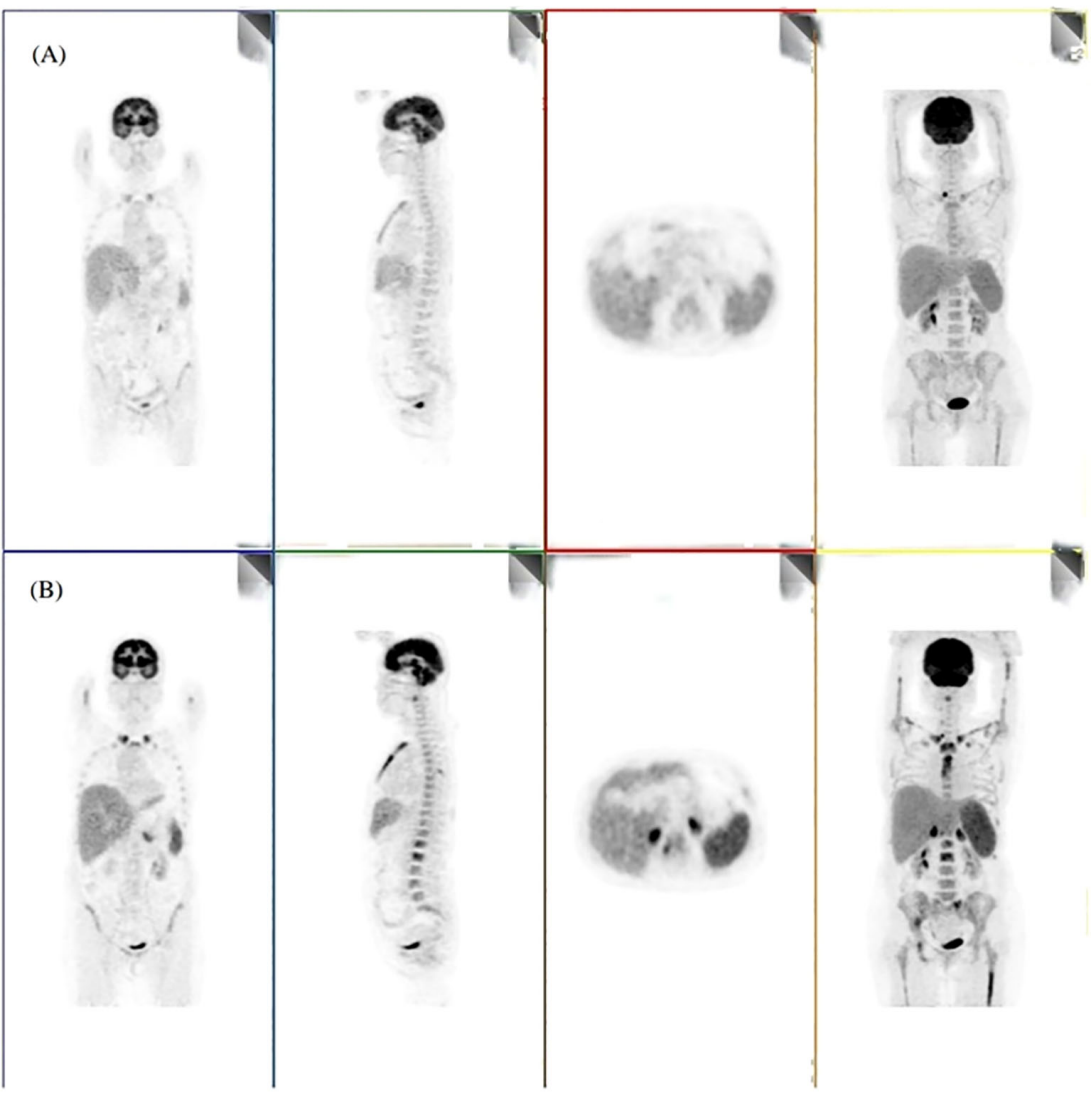
Comparative whole-body positron emission tomography/computed tomography (PET/CT) imaging at baseline diagnosis and post-treatment complete metabolic response (CMR). **(A)** Follow-up whole-body PET/CT maximum intensity projection (MIP) image obtained 12 days after the clinical onset of hemophagocytic lymphohistiocytosis (HLH) progression, after 3 cycles of pegaspargase, gemcitabine and oxaliplatin (P-GemOx) chemotherapy. This scan confirmed CMR: all previously identified hypermetabolic lymphoma lesions were completely resolved, with a Deauville score of 1 (per the international Lugano classification for lymphoma response assessment), and no radiological evidence of tumor progression or recurrence. **(B)** Baseline whole-body PET/CT MIP image obtained at initial diagnosis, showing diffuse abnormally increased metabolism in the systemic bone marrow (maximum standardized uptake value, SUVmax 10.0), irregular hypermetabolic lesions in bilateral adrenal glands (SUVmax 9.7), a hypermetabolic soft tissue lesion in the right ethmoid sinus (SUVmax 7.2), and inflammatory changes in the back subcutaneous lesion, all consistent with extensive extranodal infiltration of extranodal NK/T-cell lymphoma (ENKTCL). PET/CT, positron emission tomography/computed tomography; CMR, complete metabolic response; MIP, maximum intensity projection; P-GemOx, pegaspargase, gemcitabine and oxaliplatin; HLH, hemophagocytic lymphohistiocytosis; SUVmax, maximum standardized uptake value; ENKTCL, extranodal NK/T-cell lymphoma.

Pathological evaluation confirmed the diagnosis. Bone marrow aspiration and biopsy showed active hyperplasia (G/E=1.75:1) and markedly hypercellular marrow (≈80% cellularity), with 11.5% abnormal lymphocytes and 1.5% hemophagocytic histiocytes. Flow cytometry identified 24.1% abnormal lymphocytes with immunophenotype: sCD3^-^ cCD3^+^ CD56^+^ CD94^+^ CD2^+^ CD7^+^ CD5^-^ CD16^-^ CD4^-^ CD8^-^ CD161^-^ CD158a/b/e^-^ NKG2A^+^ NKG2C^-^ consistent with T-cell origin with NK markers. Immunohistochemistry confirmed diffuse infiltration of CD3^+^ CD7^+^ and CD56^+^ lymphocytes, consistent with lymphoma infiltration. A biopsy of the back mass, conducted seven days after admission, demonstrated diffuse infiltration of atypical lymphocytes in the dermis with focal ulcer formation. Immunohistochemistry was positive for CD3, CD7, CD56, and TIA-1, with Ki67 proliferation index > 90%. *In situ* hybridization was positive for EBV-encoded RNA (EBER), confirming ENKTCL. Baseline whole-body PET/CT performed ten days after admission revealed irregular thickening of the bilateral adrenal glands (SUVmax 9.7), diffuse hypermetabolic lesions throughout the systemic bone marrow (SUVmax 10.0), and a soft tissue lesion in the right ethmoid sinus (SUVmax 7.2), all consistent with lymphoma infiltration. Additionally, post-biopsy inflammatory changes were observed in the back subcutaneous lesion (**Figure 1**).

Final diagnoses were: 1) Extranodal NK/T-cell lymphoma, extra-nasal type, stage IVB (NRI 5 points, very high risk; PINK-E 3 points, high risk); 2) Secondary HLH. The patient met 6 of the 8 diagnostic criteria outlined in the HLH-2004 protocol (**Table 1**). Differential diagnosis systematically ruled out infection-associated HLH (negative pathogen testing, ineffective anti-infection therapy, autoimmune disease-associated. HLH (no clinical features, negative autoantibodies) and other hematologic malignancies (flow cytometry and IHC confirmed NK/T-cell origin).

**Table 1:** HLH-2004 diagnostic criteria and HScore fulfilled by the patient at baseline.

Category	HLH-2004 diagnostic criteria	Patient’s result	Fulfilled
**Molecular Diagnostic Criterion**	Molecular diagnosis consistent with HLH	No HLH-related gene mutations	No
**8 Clinical Diagnostic Criteria (≥5 items fulfilled for diagnosis)**	1.Fever ≥38.5°C	Intermittent high fevers with a peak temperature of 39°C for >2 weeks	Yes
	2.Splenomegaly	No splenomegaly	No
	3. Cytopenias (≥2 lineages)	Hb 90g/L, Plt 51×10^9^/L,	Yes
	4. Hypertriglyceridemia/hypofibrinogenemia	TG 3.03 mmol/L, Fibrinogen 1.3 g/L	Yes
	5. Hemophagocytosis in bone marrow	Present on bone marrow biopsy	Yes
	6. Low/absent NK-cell activity	NK activity 15.59% (normal)	No
	7.Ferritin ≥500μg/L	Ferritin 8287 ng/mL	Yes
	8.Soluble CD25 ≥2400 U/mL	sCD25–24227 U/mL	Yes
**Diagnostic Summary**	**-**	**6/8 clinical criteria fulfilled**	
**HScore Validation**	**Baseline: 233 points;** **progression: 290 points**	**Corresponding HLH probability: >90%;** **Corresponding HLH probability: >99%**	**Confirmatory**

HLH, hemophagocytic lymphohistiocytosis; sCD25, soluble CD25. Normal reference ranges (Huizhou First Hospital): Hb 115–150 g/L; Plt 125-350×10^9^/L; WBC 3.5-9.5×10^9^/L; Neutrophils 1.8-6.3×10^9^/L; Ferritin 13–150 ng/mL; TG 0-1.7 mmol/L; Fibrinogen 2–4 g/L; sCD25 223–710 U/mL.

The patient was definitively diagnosed with secondary HLH according to the HLH-2004 international guideline, as she fulfilled 6 out of 8 clinical diagnostic criteria in the absence of positive molecular diagnostic findings.

Bold values indicate the core HLH-2004 diagnostic criteria met by the patient and the key HScore results used for confirmatory diagnosis of HLH.

The patient’s treatment was divided into two sequential phases: HLH control and lymphoma-directed chemotherapy, with continuous multidisciplinary evaluation and informed decision-making throughout. HLH induction therapy using the DEP regimen was initiated 14 days after admission: etoposide 0.15 g intravenously once weekly, pegylated liposomal doxorubicin 40 mg intravenously on day 1, and methylprednisolone 120 mg intravenously daily on days 1-3, followed by gradual dose tapering. After induction therapy, the patient’s fever resolved, and her hematological, coagulation, and liver function parameters gradually normalized; however, she did not achieve complete remission (CR) or partial remission (PR) of HLH according to the HLH-2004 international response criteria. Three cycles of standard P-GemOx chemotherapy for ENKTCL were administered 2 weeks after HLH induction, with a three-week interval between cycles: pegaspargase 3750 IU intramuscularly on day 1, gemcitabine 1.6 g intravenously on days 1 and 8, and oxaliplatin 200 mg intravenously on day 1. The patient completed all three cycles, experiencing grade 2 myelosuppression and grade 1 gastrointestinal adverse events, which resolved with supportive care. Notably, international guidelines recommend allo-HSCT as consolidation therapy for high-risk ENKTCL with concurrent HLH. Our treatment team repeatedly discussed allo-HSCT with the patient and her family in August, September, and October 2024, including its necessity, procedure, associated costs, and potential risks (severe infection, graft-versus-host disease, organ toxicity, transplant-related mortality). The patient declined allo-HSCT due to concerns about her daughter needing to resign for long-term care, high medical costs, and transplant-related risks, with full informed understanding of the disease’s extremely high recurrence risk.

She was readmitted 4 weeks after the third cycle for the planned fourth cycle of treatment, with elevated aminotransferase levels and low fibrinogen, which were managed with liver protection therapy and fibrinogen supplementation. On day 3 of readmission, she developed a sudden recurrent high fever. Laboratory evaluation 5 days later confirmed HLH progression: ferritin increased from 2,416 ng/mL to 11,552 ng/mL, triglycerides rose from 3.96 mmol/L to 5.36 mmol/L, and plasma EBV-DNA reached 1.64 × 10^5^ copies/mL by the time of HLH progression diagnosis (Day 109). Her HScore at this time was 290 points. Critically, repeat whole-body PET/CT performed 12 days after the onset of HLH progression showed complete resolution of all previously hypermetabolic lymphoma lesions (Deauville score 1, consistent with CMR), with no radiological evidence of tumor progression. Repeat bone marrow aspiration and biopsy were not performed due to the patient’s extremely poor clinical status, rapidly progressive coagulopathy, thrombocytopenia (nadir platelet count 22 × 10^9^/L), high bleeding risk, and refusal of invasive procedures by her legal surrogate decision-makers.

Sequential salvage therapies were administered. Two cycles of DEP chemotherapy, given 3 days after the diagnosis of HLH progression, resulted in transient clinical improvement, with ferritin levels declining to 6,732 ng/mL; however, the response was short-lived. Ruxolitinib 10 mg daily was initiated 24 days after progression onset, escalated to 15 mg twice daily 1 week later, and combined with dexamethasone. Despite this, ferritin levels began rising again 2 weeks after the dose escalation. Ruxolitinib was discontinued 5 weeks after progression diagnosis, and the fourth cycle of P-GemOx was administered with full informed consent. The rationale included concurrent EBV-DNA elevation suggesting potential subclinical tumor reactivation, only a transient response to prior salvage therapies, and a multidisciplinary consensus that lymphoma-directed chemotherapy was the only potential upstream intervention. 1 week after the fourth P-GemOx cycle, she developed recurrent high fever, with ferritin surging to 35,635 ng/ml. Subsequent therapies, including two rounds of plasma exchange, golidocitinib, and mitoxantrone 20 mg, failed to control hyperinflammation. Ferritin peaked at 87,918 ng/mL and triglycerides at 9.6 mmol/L 6 weeks after the fourth cycle, accompanied by progressive acute respiratory distress syndrome, acute renal injury, and circulatory collapse consistent with terminal HLH-related multiorgan dysfunction. She died of multiorgan failure the following day, while maintaining CMR on PET/CT.

**Figure 2:**
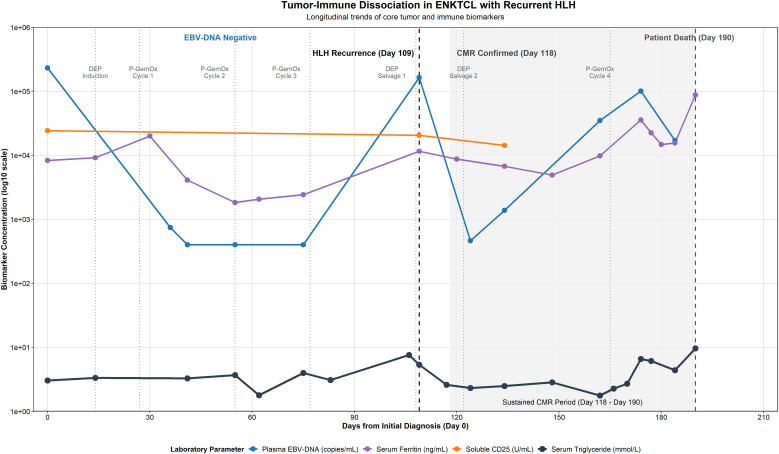
Dynamic longitudinal trends of tumor burden and immune activation biomarkers in a patient with stage IVB extranodal natural killer/T-cell lymphoma (ENKTCL) and secondary hemophagocytic lymphohistiocytosis (HLH). The X-axis represents days from baseline admission (Day 0, defined as the day of initial hospital admission), and the Y-axis shows biomarker concentrations on a log10 scale to accommodate the wide dynamic range of values across different analytes. Lines depict serial changes of four core biomarkers: blue line = plasma Epstein-Barr virus (EBV) DNA load (copies/mL, tumor burden biomarker); purple line = serum ferritin (ng/mL, HLH disease activity biomarker); orange line = serum soluble CD25 (sCD25, U/mL, systemic immune activation biomarker); black line = serum triglyceride (mmol/L, HLH diagnostic and activity biomarker). The gray shaded area indicates the period of sustained complete metabolic response (CMR), which was confirmed by whole-body positron emission tomography/computed tomography (PET/CT) on Day 118. Vertical black dashed lines mark the time of HLH progression onset (Day 109) and patient death due to HLH-related multiple organ failure (Day 190), respectively. Vertical gray dotted lines indicate the timing of all key treatment events, including initial HLH induction therapy, four cycles of P-GemOx chemotherapy, and two cycles of salvage DEP chemotherapy after HLH recurrence. Notably, progressive elevation of HLH activity and immune activation biomarkers was observed despite sustained radiological CMR of the primary lymphoma, demonstrating the core “tumor-immune dissociation” phenomenon defined in this report: aberrant immune activation and HLH progression occurred independently of radiologically detectable tumor burden. ENKTCL, extranodal natural killer/T-cell lymphoma; HLH, hemophagocytic lymphohistiocytosis; EBV, Epstein-Barr virus; sCD25, soluble CD25; CMR, complete metabolic response; PET/CT, positron emission tomography/computed tomography.

Serial dynamic monitoring of dual-track tumor and immune biomarkers throughout the entire disease course (**Figure 2**) revealed a striking and previously unreported phenomenon of “tumor-immune dissociation.” Plasma EBV DNA, the core molecular biomarker of lymphoma tumor burden, turned negative after 1 cycle of P-GemOx chemotherapy (Day 41) and remained below the lower limit of detection until Day 75. This finding was consistent with the subsequent radiologically confirmed complete metabolic response (CMR) on Day 118. In stark contrast, HLH-specific activity biomarkers (ferritin, triglycerides) and the systemic immune activation biomarker soluble CD25 (sCD25) remained markedly abnormal, with laboratory-confirmed HLH progression on Day 109—despite no radiological evidence of lymphoma recurrence, progression, or new hypermetabolic lesions on whole-body PET/CT. This dissociation between complete radiological tumor control and uncontrolled immune hyperactivation persisted until the patient’s terminal stage, with ferritin peaking at 87,918 ng/mL and triglycerides at 9.6 mmol/L on Day 190 (**Table 2**).

**Table 2:** Dynamic changes of core dual-track biomarkers across key clinical time points.

Relative time point	Plasma EBV-DNA (copies/mL)	Ferritin (ng/mL)	sCD25(U/mL)	Triglyceride (TG,mmol/L)	Clinical event
Day 0 (Baseline Admission)	2.32×10^5^	8287	24227	3.03	Initial diagnosis ENKTCL+HLH
Day 14	–	9167	–	3.33	Before HLH induction
Day 41	<4.00×10²	4096	–	3.27	After cycle 1 P-GemOx
Day 55	<4.00×10²	1827	–	3.68	Before cycle 2 P-GemOx
Day 75	<4.00×10²	2416	–	3.96	Before cycle 3 P-GemOx
Day 109	1.64×10^5^	11552	20565	5.36	HLH progression onset
Day 134	1.38×10³	6732	14252	2.48	Post salvage therapy
Day 162	3.48×10^4^	9808	–	1.76	Before cycle 4 P-GemOx
Day 174	1.01×10^5^	35635	–	6.56	Refractory HLH progression
Day 190	–	87918	–	9.6	Terminal stage

CMR, complete metabolic response; ENKTCL, extranodal NK/T-cell lymphoma; HLH, hemophagocytic lymphohistiocytosis; sCD25, soluble CD25; “-”, not tested. Same normal reference ranges as **Table 1**.

Serial sCD25 testing was conducted only at three key time points due to send-out testing requirements, high out-of-pocket costs without insurance coverage, and the patient and family’s decision to decline routine serial monitoring. From the patient and family’s perspective, the rapid progression of HLH despite apparent radiological tumor control was an unexpectedly devastating outcome. The patient’s legal surrogate decision-makers provided written informed consent for the publication of this de-identified case report, expressing the hope that this clinical experience could help identify early warning signs of HLH progression in future high-risk patients.

## 3 Discussion

ENKTCL patients with concurrent HLH have a dismal clinical prognosis, with HLH recognized as the leading cause of early mortality in this population (5). However, current clinical studies and guidelines primarily focus on the diagnosis and initial management of HLH at the time of ENKTCL diagnosis. The clinical characteristics, underlying pathogenesis, and early identification strategies for HLH progression in patients who have achieved CMR remain poorly understood, representing a significant gap in current follow-up protocols. In this case, the patient achieved radiologic CMR after standard treatment but subsequently developed recurrent and ultimately refractory HLH progression without radiologic or pathological evidence of viable tumor progression. This presents a clear “tumor-immune dissociation” phenomenon that cannot be explained by the traditional view that tumor burden is the sole driver of M-HLH. We interpret the underlying immunological mechanisms based on existing authoritative studies, which are fully consistent with our clinical data.

First, persistent antigen presentation may drive this dissociation. ENKTCL is an EBV-driven lymphoma, and previous studies have demonstrated that EBV-related antigens released by tumor cells during chemotherapy can be persistently presented by antigen-presenting cells *in vivo*, even after all viable tumor cells have been cleared (6). In this patient, these persistent antigens may have continuously activated EBV-specific CD8^+^ cytotoxic T cells, contributing to excessive secretion of IFN-γ, TNF-α, and other pro-inflammatory cytokines during HLH progression. IFN-γ, the core upstream cytokine of HLH, directly drives macrophage overactivation and M1 polarization, amplifying the cytokine storm without viable tumor cells (7, 8). This mechanism aligns with our clinical data: at HLH progression, the patient had detectable plasma EBV-DNA without any hypermetabolic tumor lesions on PET/CT, suggesting that persistent antigenic stimulation, rather than viable tumor cells, may have driven immune activation (9, 10). Importantly, we acknowledge that subclinical EBV reactivation or microscopic lymphoma infiltration undetectable by PET/CT cannot be fully ruled out as concurrent trigger.

Second, pre-existing immune dysregulation combined with sustained inflammatory stimulation provides a rational explanation for HLH progression despite radiologic tumor clearance. The core pathological feature of secondary HLH is the loss of homeostatic control over innate and adaptive immune responses, resulting in unrestrained macrophage activation and a self-sustaining cytokine storm (5, 11). The patient’s initial HLH episode confirmed a profound impairment in the negative regulation of inflammatory responses, a known risk factor for recurrent cytokine storms, even after the initial triggering insult has resolved (5, 11). Even after CMR was achieved, persistent EBV antigen presentation provided chronic pro-inflammatory stimulation, while multi-cycle chemotherapy induced subclinical tissue damage and the release of endogenous inflammatory mediators, further lowering the activation threshold of circulating monocytes and tissue-resident macrophages (12). Together, these factors were sufficient to trigger uncontrolled macrophage activation and HLH onset independent of radiologically detectable tumor burden (13, 14). The HScore, widely validated for adult HLH diagnosis and activity monitoring, increased from 233 at baseline to 290 at HLH progression onset, then increased further to 300 at the terminal stage, consistent with worsening immune hyperactivation and disease severity.

Third, the self-sustaining nature of the cytokine storm, mediated by the JAK/STAT signaling axis, can drive progression to refractory HLH independent of the initial tumor trigger. The JAK/STAT pathway is the core signaling axis mediating the pro-inflammatory effects of HLH-associated cytokines (15–17). Once initiated, continuous cytokine secretion sustains JAK/STAT activation, amplifying inflammation into a self-perpetuating vicious cycle independent of the initial trigger. This explains the refractory HLH progression after tumor clearance and the limited efficacy of ruxolitinib and golidocitinib (15). Serum triglyceride, a core HLH-2004 criterion, and its dynamic changes in this patient closely mirrored HLH disease activity. It remains a readily available, low-cost biomarker for real-time HLH activity monitoring in resource-limited clinical settings.

In this case, sCD25 was used as a key biomarker of immune activation but was measured only at three key time points, reflecting real-world barriers to routine sCD25 surveillance, including limited access, high out-of-pocket costs, and lack of national standardization. These barriers must be addressed to integrate sCD25 into routine surveillance for high-risk ENKTCL patients. The primary strength of this case report lies in its detailed description of refractory HLH progression despite sustained CMR in a high-risk ENKTCL patient. It introduces the concept of “tumor-immune dissociation” and fills a critical gap in the literature, as current guidelines predominantly focus on tumor burden monitoring during ENKTCL follow-up. This study has inherent limitations as a single case report. Additional limitations include the inability of PET/CT to detect microscopic disease, lack of repeat bone marrow evaluation at HLH progression, limited serial sCD25 testing, unconfirmed mechanistic causality, and no tumor-targeted NGS due to rapid clinical deterioration and family refusal. Two pathogen NGS tests ruled out infection: 1) Aug 12, 2024: Only EBV detected (7.61×10³ copies/mL); 2) Dec 2, 2024: EBV (high confidence) and commensal *Bacteroides fragilis* (non-pathogenic). Therefore, our findings are hypothesis-generating and require validation in larger cohorts.

Current follow-up protocols for ENKTCL only focus on tumor burden monitoring, ignoring immune activation and HLH activity biomarkers. This case clearly demonstrates that HLH can develop even after CMR, due to persistent immune dysregulation independent of radiologically detectable tumor burden. For high-risk ENKTCL patients with a history of HLH, serial monitoring of sCD25, ferritin, and triglycerides should be added to post-remission follow-up to identify early warning signs of HLH progression. Preemptive intervention before irreversible cytokine storm formation, as well as early allo-HSCT evaluation for eligible patients, is key to improving outcomes in ENKTCL-related HLH.

## 4 Conclusion

This case illustrates a patient with stage IVB ENKTCL who developed recurrent refractory HLH progression despite achieving radiologic CMR after standard chemotherapy. This reveals a critical “tumor-immune dissociation” phenomenon that challenges current follow-up paradigms focused solely on radiologically detectable tumor burden. The case highlights the importance of serial monitoring of immune activation and HLH disease activity biomarkers, in addition to routine tumor burden monitoring in high-risk ENKTCL patients with a history of HLH. It also provides preliminary clinical clues for exploring early identification and preemptive intervention strategies for ENKTCL-related HLH. Early evaluation of allo-HSCT should be strongly recommended for this high-risk population to reduce the risk of HLH progression and improve survival. Further studies should address the accessibility and standardization of immune biomarker testing.

## Data Availability

The original contributions presented in the study are included in the article/supplementary material, further inquiries can be directed to the corresponding author/s.
